# Stimulated Brillouin Scattering Microscopic Imaging

**DOI:** 10.1038/srep18139

**Published:** 2015-12-22

**Authors:** Charles W. Ballmann, Jonathan V. Thompson, Andrew J. Traverso, Zhaokai Meng, Marlan O. Scully, Vladislav V. Yakovlev

**Affiliations:** 1 Texas A&M University, College Station, TX 77843-4242; 2 Princeton University, Princeton, NJ 08544; 3 Baylor University, Waco, TX 76798.

## Abstract

Two-dimensional stimulated Brillouin scattering microscopy is demonstrated for the first time using low power continuous-wave lasers tunable around 780 nm. Spontaneous Brillouin spectroscopy has much potential for probing viscoelastic properties remotely and non-invasively on a microscopic scale. Nonlinear Brillouin scattering spectroscopy and microscopy may provide a way to tremendously accelerate the data aquisition and improve spatial resolution. This general imaging setup can be easily adapted for specific applications in biology and material science. The low power and optical wavelengths in the water transparency window used in this setup provide a powerful bioimaging technique for probing the mechanical properties of hard and soft tissue.

Brillouin scattering originates from a non-elastic light interaction with acoustic waves in a medium that are generated by thermodynamic fluctuations. The scattered light is shifted in frequency according to the relation^1^


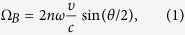


where *n* is the index of refraction, *ω* is the frequency of the incident light, 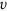 is the speed of sound in the material, *c* is the speed of light in vacuum, and *θ* is the angle between the incoming and backscatterd light. The speed of sound for a material depends on properties such as compressibility, shear modulus and density for solids, and pressure, density, temperature, composition and heat capacity for non-ideal gases and liquids. Some of these properties listed above are not easily determined by spectroscopy techniques that interact with atomic or molecular energy levels. Brillouin scattering provides a powerful method to assess these properties. In addition, fluorescent methods often require wavelengths in a specific wavelength range, but Brillouin scattering, similar to Raman scattering spectroscopy, will always give a frequency shift. This allows the option for laser sources that are, for example, cheaper and/or have frequencies that scatter (Rayleigh and Mie) less in the material of interest. Since Brillouin scattering is a label-free, frequency independent method, it opens many new possibilities in biosensing and imaging. Brillouin scattering offers a possible route for deep tissue imaging by selecting a longer wavelength where lossy (Rayleigh and Mie) scattering in tissue is relatively small. Furthermore, the generated signal, being very close in wavelength to the incident light, will also have minimal lossy scattering and transmit out of the sample for detection.

The first published theoretical work predicting the scattering of photons by acoustic phonons was by Brillouin in 1922^2^ and was experimentally verified in crystals and liquids in 1930 by Gross^3^. Over the years, spontaneous Brillouin scattering has been applied to many complex materials such as muscle fibers^4^, bone tissue^5^, eyes^6^, spider silks^7^, thin films^8^ and quantized spin waves^9^. A few physical properties of materials that spontaneous Brillouin has been applied to are tensil and compresive strain^10^, temperature^11^, elastic moduli^12^,^13^, bulk viscocity^14^, acoustic velocity, refractive index, and phonon lifetime^15^. Brillouin scattering can be performed using surface acoustic waves as well and not just in bulk material. Surface Brillouin scattering has been used to characterize very hard films^16^ and thin metal films^17^. Brillouin scattering has also been proposed for remote sensing^18^ such as in Brillouin-LIDAR^19^,^20^.

Many instrumentation improvements have recently been made for spontaneous Brillouin experiments^21^,^22^,^23^, particularily for biological applications. Since Brillouin scattering is a non-contact, label free method, it has seen many applications in biology for the measurement of properties that would be difficult to measure with other methods. The internal mechanical properties of a single biological cell have been reported using GHz ultrasound imaging induced by ultrashort laser pulses^24^,^25^, as well as the elastic properties of viruses, which have been assessed using conventional Brillouin spectroscopy^26^. Several studies have been initiated to observe changes in soft tissue due to injury or disease^27^,^28^,^29^. Confocal Brillouin and Raman microscopy has been combined to give exciting flexibility for a large number of applications, with the ability to measure many properties simultaneously^30^,^31^. Recently, we have demonstrated simultaneous imaging spontaneous Brillouin and Raman microscopy^32^; clearly, the same approach can be extended to nonlinear Brillouin and Raman microscopes, which potentially offer better spatial resolution and faster acquisition rate imaging.

Stimulated Brillouin scattering was first observed experimentally by Chiao *et al.* in 1964^33^. Spontaneous Brillouin scattering becomes stimulated Brillouin scattering when a second counterpropagating beam (with frequency detuned from the first beam by 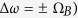 causes beating between the fields and enhances the sound wave. This second beam is either a separate probe beam (the method used in this work), or if the incident beam has enough intensity such that backscattered spontaneous Brillouin becomes appreciable, this backscattered light will beat with the incident beam. A striking advantage stimulated Brillouin has over spontaneous Brillouin is that the effeciency of conversion can be much higher, and with sufficient intensities, can approach 100%^15^. In addition, stimulated Brillouin is not encumbered by the large spontaneous Rayleigh peak, the spectral resolution of the obtained spectrum depends only on the linewidth of the lasers used, and the spectral range is limited only by the pump tuning range^34^. All properties that can be measured with spontaneous Brillouin spectroscopy can also be measured with stimulated Brillouin scattering spectroscopy. However, there are limitations to the use of stimulated Brillouin scattering such as power and geometry. For instance, high power lasers with tunable narrowband radiation are not routinely available, and a special arrangement is needed to allow for a counterpropagating probe beam. In addition, laser stability is a significant concern, especially when using a pump-probe setup, and the alignment of the two beams must be very precise for spatial overlap in the focal region. Laser stability and tunability can be overcome by the use of high-speed EOMs^35^, in this way the beams are self referenced and drift is no longer a concern.

In the literature, most studies and applications of stimulated Brillouin scattering have been done in optical fibers. Very little has been done to apply it to biology, making it a relatively unexplored subfield of stimulated Brillouin scattering. For spontaneous Brillouin, two^36^ and three^6^ dimensional imaging has been done, but, to our knowledge, imaging has not been done previously with stimulated Brillouin scattering. Going to stimulated Brillouin has the benifits discussed above, especially high conversion effeciency, wide tuning abilities and no spontaneous Rayleigh peak.

In this work, we apply stimulated Brillouin scattering in a proof-of-principle experiment to perform 2D imaging. A brief theoretical description is given to provide an order of magnitude estimation of signal strength to be compared to the experimental data. Finally, the image results are shown and methods to improve and extend the current setup for more challenging applications are discussed.

## Theory

**Figure 1:**
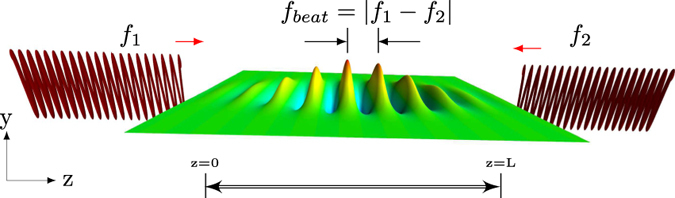
Conceptual picture illustrating stimulated Brillouin scattering. The two fields are incident from the left and right at frequencies f_1_ and f_2_ respectively. In the center, the waves in the medium represent the density variations (acoustic waves). L is the total interaction length in the sample. (Figure graphic generated in Mayavi2^42^).

Stimulated Brillouin scattering (SBS) can be treated with a general theoretical model of light scattering from inhomogeneous thermodynamic fluctuations caused primarily by fluctuations in pressure (Brillouin) and temperature (Rayleigh) (see, e.g., Boyd^15^ Ch. 9). A conceptual picture of stimulated Brillouin for couterpropagating pump and probe beams is shown in Fig. 1. The counterpropagating pump and probes beams with center frequencies at 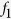 and 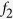, respectively, beat together in the medium, which will generate and amplify the sound wave in the medium which oscillates at the beat frequency 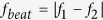. Efficient stimulated Brillouin scattering of the beams will occur only when 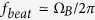. This nonlinear (stimulated) interaction of light with the medium is due primarily to electrostriction and absorption, however absorption is a small effect for SBS except for lossy optical media^15^.

In the following derivation and results, we assume that 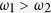, but it is easily applied to the other case. Starting with the equation of motion for a pressure wave^1^ and using the approximations that phonon propagation distance is small compared to distance that the source term varies dramatically, and assuming steady state conditions, we easily obtain an expression for the acoustic waves. This expression is then used as the fluctuating dielectric constant in the expression for the polarization of the medium. This expression and the expressions for the optical fields are then used in the wave equation to determine the field propagation. After making the slowly-varying amplitude approximation, using steady state conditions and setting 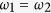 (except for frequency difference terms) since 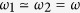, the equations for the spatial rate of change of the intensities of the two fields are


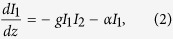



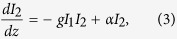


where *α* is the optical absortion coefficient and g is the gain factor for SBS. In equation 3, the second term on the RHS has a plus sign since I_2_ is propagating in the negative z direction (see Fig. 1). The gain factor g can be broken up into electrostrictive and absorptive factors as


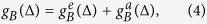


where 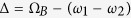. For optical materials with low absorption 
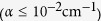
, electrostriction is the dominant effect for SBS generation. For our proof-of-principle experiment, we used water, which has an absorption coefficient of 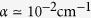 at 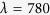 nm^37^. Using equations 2 and 3, we can derive an expression in the very weak probe and undepleted pump limit for the intensity of the probe after the interaction. For a weak probe and for pump intensities sufficiently low such that spontaneous Brillouin from the pump is weaker than the probe, 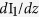 is very small. Therefore, in this limit, I_1_ is approximately constant in equation 3. In addition, *α* is very small in our case, so the second term on the RHS of equation 3 will be ignored. Solving this equation (integrating from L to z) with 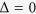 yields


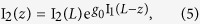


where 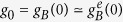 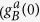 has been neglected since it is very small for our case). Equation 5 will be used in the results/discussion section to derive the expected order of magnitude for the signal. The exact solutions to equations 2 and 3 (with 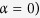 leads to a transcendental equation and must be numerically solved. However, looking at equation 3 tells us that increasing the intensity of either or both of the lasers increases the rate of increase of the signal (plotted numerical solutions in^15^ show this clearly). The expression for the Brillouin shift is given in equation 1, and the Brillouin linewidth (FWHM) can expressed as^15^


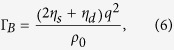


where 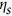 is the shear viscocity coefficient, 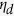 is the dilational viscocity coefficient, 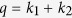 is the phonon wavenumber, and 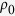 is the average density of the material. The Brillouin gain, 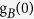 for water is 0.048 m/GW, which is a factor of 3 to 5 less than many alcohols, ketones, or hydrocarbons. Water was chosen for our experiment because the volatility of water is lower than the previous mentioned compounds and was suitable for a long scan with little evaporation.

## Results and Discussion

**Figure 2:**
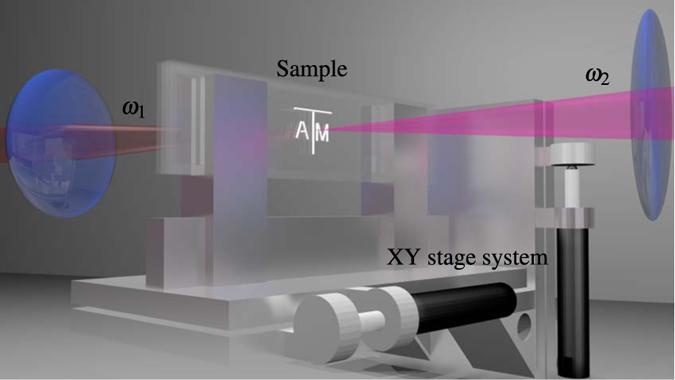
Schematic of the imaging setup. For illustration, the pump and probe beams are red 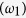 and magenta 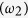, respectively. Two glass slides are sandwiched together with 1 mm of separation between them and sealed to form a cell that was filled with water. The entire sample was mounted on a motorized xy stage system. See Methods section for further details.

A close-up schematic of the imaging setup is shown in Fig. 2. A fixed probe laser and a tunable, counterpropagating pump laser are focused inside a sample to generate the SBS signal. The sample is mounted on a computer controlled xy stage system and scanned across the overlapped focal region of the two counterpropagating beams.

**Figure 3:**
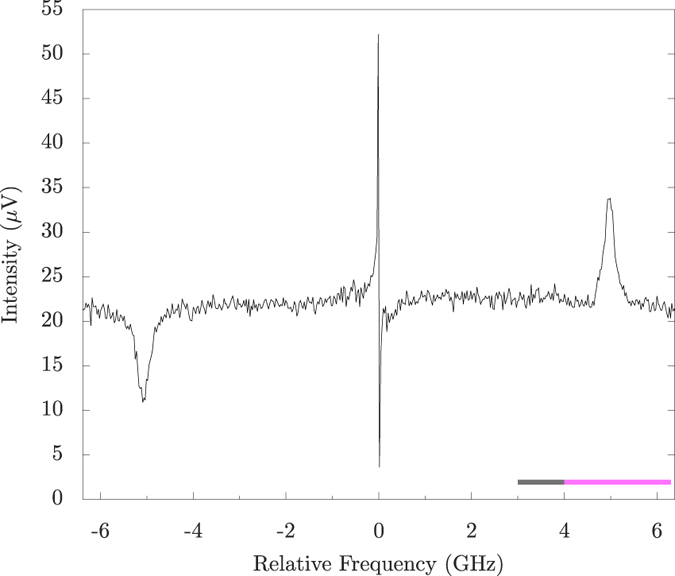
SBS spectrum of water. The loss and gain peaks on the left and right, respectively, are from SBS while the center feature is from absorptive stimulated Rayleigh scattering. The Brillouin frequency shift is measured to be 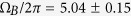 GHz with a Brillouin linewidth of 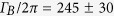 MHz. The grey and magenta bars at the bottom indicate the frequency ranges used for the background (Fig. 4b) and signal (Fig. 4c), respectively, of the image scans.

Figure 3 shows a SBS spectrum of H_2_O (T = 20.5 °C, reverse osmosis filtered) at one spot on the sample. The scan was performed using a ~26 MHz step size. Using the formulas above, the Brillouin shift and linewidth are calculated to be 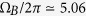 GHz and 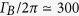  MHz (using^38^ for viscocity coefficients), and are measured to be 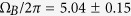  GHz and 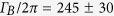  MHz. This is in agreement with previous experiments^15^. The dip on the left (at ~−5 GHz) and peak on the right (at ~5 GHz) of zero are the stimulated Brillouin loss and gain peaks, respectively, caused by the first term on the RHS of equation 4. The center feature is caused by absorptive stimulated Rayleigh scattering. The signal to noise for this experiment is calculated to be ~15 for SBS in Fig. 3. For an order of magnitude calculation of the expected signal, we can use equation 5 (since the approximations for deriving equation 5 are valid) for the signal after the interaction 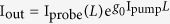, where g_0_ is the max SBS gain and L is the total interaction length. For our case, g_0_ = 0.048 m/GW, L 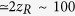 *μ*m (*z*_*R*_ is the Rayleigh length; the signal will come almost entirely where the intensity is the largest), and 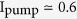 GW/m^2^. This yields 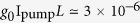. Therefore, 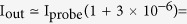

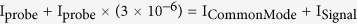
. From this we see that the maximum SBS signal going into the detector is about 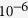 of the total probe power 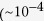 W) going into the detector. Therefore this SBS signal is 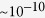 W. The shot noise limit of the balanced detector used is ~120 dB, and the best common mode rejection ratio is close to this (~118 dB), therefore the weak signal 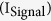 will experience ~10^5^ gain with respect to gain of the common mode probe signal 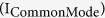. The detector has 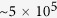 V/W gain which amplifies the signal to ~10^−5^ V. As can be seen in Fig. 3, this is about the expected order of magnitude.

**Figure 4:**
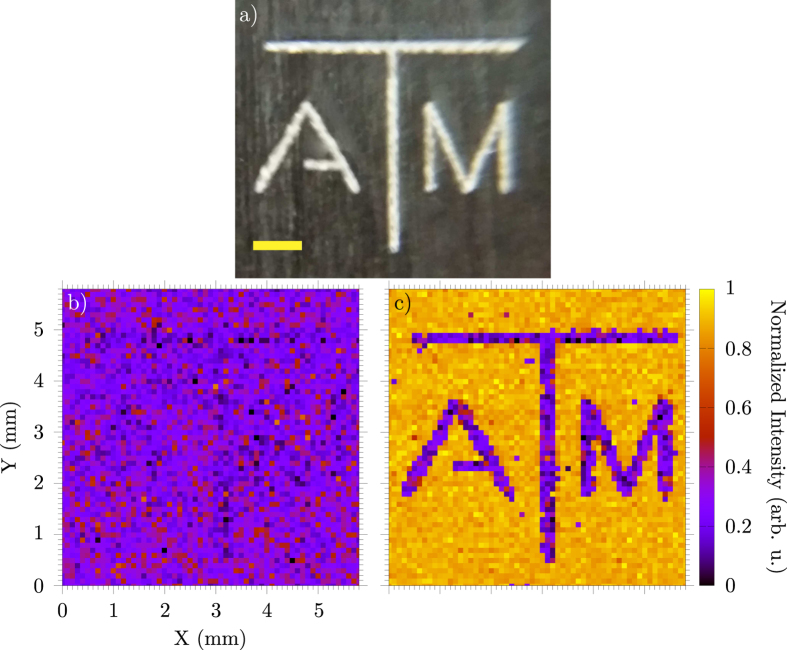
(**a**) A picture of the image etched on a clear microscope slide (grey background is the table, yellow scale bar is 1 mm in horizontal direction). The etched lines are ~250 *μ*m wide. Scan of the image in water without (**b**) and with (**c**) SBS. The step size is 100 *μ*m in each dimension. In (**b**), the resulting image obtained when the frequency scan (per pixel) excludes the SBS gain peak by scanning the ~3–4 GHz frequency range indicated by the grey bar in Fig. 3. In (**c**), the same image scan but with the SBS signal from water obtained by scanning the ~4–6.3 GHz region indicated by the magenta line in Fig. 3. The signal in (**c**) is generated everywhere except where the glass is etched. The signals in (**b**,**c**) are normalized with the maximum of (**c**).

In our experiment, we obtained a 2D image with SBS by scanning a limited region in frequency covering the gain peak for each pixel in the image. For Fig. 4c, each pixel scan took ~1 min to obtain with the frequency scan range from ~4–6.3 GHz (magenta bar in Fig. 3). The spatial scan step size was 100 *μ*m in both dimensions. Figure 4b is a comparison image that was taken with frequency scans from ~3–4 GHz (grey bar in Fig. 3), so the SBS signal is not present. The sample consisted of two glass slides separated by 1 mm with water sealed between them. On one slide, the logo was etched on the inside of the glass by a CO_2_ laser cutting machine (Fig. 4a). The lines making up the letters are ~250 *μ*m wide. This etch frosts the glass and attenuates the probe beam when scanning over it. Therefore, the signal from water is everywhere except where the logo blocks the probe. By performing a frequency scan (scanning range ~2.3 GHz with ~50 MHz steps) for each pixel, a good signal to noise ratio was obtained to discern between signal and backscattered light. In addition, these scans allow for the small drift in the lasers over the duration of the scan without having to lock them.

The present imaging configuration is time consuming and would not be practical for real world applications. Fortunately, this setup can be easily modified to perform similar imaging at much higher aquisistion speeds with improved spatial and spectral resolution. For example, a tremendous improvement in frequency stability and low acquisition time can be achieved by implementing a high frequency EOM to create a Stokes/anti-Stokes pair^35^ and a virtually imaged phased array (VIPA) with a CCD for faster signal acquisition. In the theory section, we discussed that increasing the intensity of either or both fields will yield a stronger signal. Therefore, using either more powerful CW lasers or using pulsed lasers would yield a stronger signal. In addition, photons at wavelengths longer than 1000 nm greatly reduce optical damage and penetrate biological samples well due to low absorption and small Rayleigh and Mie scattering cross sections. However, optical damage is also inversly proportional to the pulse length due primarily to multi-photon effects^39^,^40^,^41^. Therefore, care should be taken when using short-pulsed (pulse durations of a few picoseconds or less) lasers, especially on biological samples. With low power, low frequency CW lasers, such nonlinear effects are no longer a concern. In this setup, instead of generating a large signal from high power lasers, we focused on better signal sensitivity. This resulted in a measurable signal with pump and probe powers as low as 10 mW and 5.5 mW respectively. However, for many materials the SBS gain 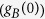 is small (compared to 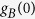 of water), making detection difficult. By switching to lasers with outputs around several hundred milliwatts, this would drastically increase our SBS signal.

## Conclusion

In conclusion, we demonstrated a two dimensional SBS imaging system. As a proof-of-principle, we obtained a two dimensional image using water as the signal medium. Our setup utilized two amplitude modulated CW diode lasers, achieving 25 and 8 mW average power on the sample from the pump and probe, respectively. Comparison between theory and experiment was made, and improvements in the experiment design for achieving a stronger signal and faster aquisition times was discussed. The methods in this paper are shown to have many benifits for biological applications and provide a unique tool by which samples may be characterized.

## Methods

**Figure 5:**
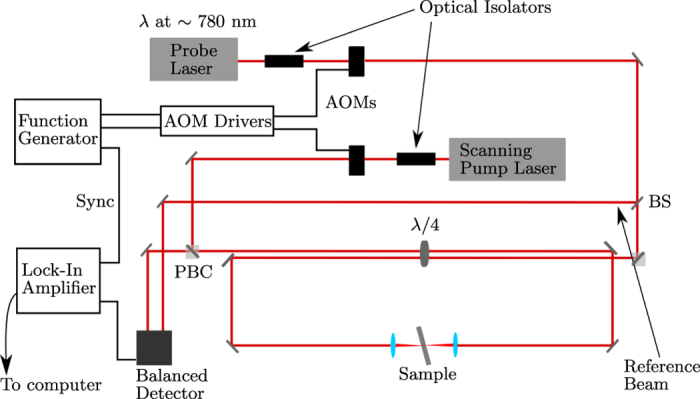
Schematic of the experimental setup. BS: Beam Splitter, PBC: Polarized Beam Cube, AOM: Acousto-optic Modulator.

In the experimental setup (Fig. 5), a ~45 mW tunable CW diode laser (Newport, Inc.; Vortex II TLB-6900) is used as the probe beam. The pump laser used is a >100 mW tunable CW diode laser (Sacher Lasertechnik; Lion TEC-520-0780-100-M). Only the pump wavelength was scanned. The probe laser was tuned to the Rb D_2_ transition 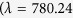 nm) and remained untouched for the rest of the experiment. The pump and probe beams were amplitude modulated (Gooch & Housego; 23080-1-LTD AOM) at 102 kHz and 2 kHz respectively. The high modulation frequency substantualy reduces the noise in the sample. The difference frequency was output to the lock-in amplifier (Stanford Research Systems; SR830 DSP) for the reference frequency and the time constant was set to 300 ms. A portion of the probe beam ~1 mW was split off after the acousto-optic modulator (AOM) using a CaF_2_ window and then attenuated before being used as the reference beam in the balanced detector (New Focus; Nirvana 2007). Both beams were passed through 50 *μ*m pinholes after the AOMs to clean up the spatial beam mode of the diode lasers. The polarization of both beams was filtered by using 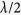 waveplates and polarizing beam cubes. After the polarizing beam cubes, the two beams were passed side by side through a 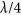 waveplate and converted into circularely polarized light in order to easily separate the two beams after the interaction. The counterpropagating pump and probe beams were then focused on the sample using 35 mm and 25.4 mm lenses respectively.

The estimated diameters at the focus for the pump and probe beams are ~4 *μ*m and ~7 *μ*m respectively. By the time the pump and probe reach the sample, the average power was 25 mW and 8 mW respectively. The sample was mounted on a xy stage system and was tilted to remove back reflections. After interaction, the beams then travelled back through the 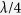 waveplate and were now rotated by 90 degrees. In this way, each beam could be removed from the beam paths at the polarization cubes with minimal loss in power. The probe pulse was then attenuated with a neutral density filter (~200 *μ*W) and sent into the balanced detector input. The output signal from the balanced detector is first sent in to a highpass filter to remove the low frequency probe modulation and then sent into the lock-in amplifier. Both lasers, the lock-in amplifier and the xy stage were connected and controlled by a computer using NI LabVIEW. All optics used had an anti-reflective coating for 780 nm.

## Additional Information

**How to cite this article**: Ballmann, C. W. *et al.* Stimulated Brillouin Scattering Microscopic Imaging. *Sci. Rep.* **5**, 18139; doi: 10.1038/srep18139 (2015).
